# The institutional context of tobacco production in Zambia

**DOI:** 10.1186/s12992-018-0328-y

**Published:** 2018-01-16

**Authors:** Ronald Labonté, Raphael Lencucha, Jeffrey Drope, Corinne Packer, Fastone M. Goma, Richard Zulu

**Affiliations:** 1 Faculty of Medicine, School of Epidemiology and Public Health, University of Ottawa, Room 205, 600 Peter Morand Cres, Ottawa, ON K1G 5Z3 Canada; 2 Faculty of Medicine, School of Physical and Occupational Therapy, McGill University, 300B Hosmer House, 3630 Promenade Sir William Osler, Montreal, QC H3G 1Y5 Canada; 3 American Cancer Society, Inc., 250 Williams Street, Atlanta, GA 30303 USA; 4 School of Medicine, Centre for Primary Care Research, University of Zambia, P.O. Box 50110, Lusaka, Zambia

**Keywords:** Framework convention of tobacco control, Tobacco, Zambia, Tobacco policy, Whole-of-government approach, Institutional actors, Intersectoral action, Economic policy

## Abstract

**Background:**

Tobacco production is said to be an important contributor to Zambia’s economy in terms of labour and revenue generation. In light of Zambia’s obligations under the WHO Framework Convention of Tobacco Control (FCTC) we examined the institutional actors in Zambia’s tobacco sector to better understand their roles and determine the institutional context that supports tobacco production in Zambia.

**Methods:**

Findings from 26 qualitative, semi-structured individual or small-group interviews with key informants from governmental, intergovernmental and non-governmental organisations were analysed, along with data and information from published literature.

**Results:**

Although Zambia is obligated under the FCTC to take steps to reduce tobacco production, the country’s weak economy and strong tobacco interests make it difficult to achieve this goal. Respondents uniformly acknowledged that growing the country’s economy and ensuring employment for its citizens are the government’s top priorities. Lacklustre coordination and collaboration between the institutional actors, both within and outside government, contributes to an environment that helps sustain tobacco production in the country. A Tobacco Products Control Bill has been under review for a number of years, but with no supply measures included, and with no indication of when or whether it will be passed.

**Conclusions:**

As with other low-income countries involved in tobacco production, there is inconsistency between Zambia’s economic policy to strengthen the country’s economy and its FCTC commitment to regulate and control tobacco production. The absence of a whole-of-government approach towards tobacco control has created an institutional context of duelling objectives, with some government ministries working at cross-purposes and tobacco interests left unchecked. With no ultimate coordinating authority, this industry risks being run according to the desire and demands of multinational tobacco companies, with few, if any, checks against them.

## Background

Zambia was one of three Sub-Saharan African countries (Kenya and Malawi being the other two) selected for a comparative study of the political economy of trade, tobacco farming and tobacco control. The countries were selected on the basis of their differences in the role of tobacco as an agricultural crop and their engagement with tobacco control strategies. Kenya has the most advanced tobacco control policies and the lowest level of tobacco as a contributor to its economy. Malawi has not ratified the World Health Organization’s Framework Convention on Tobacco Control and has the least forward movement of the three countries on tobacco control measures. Zambia falls between the two, is experiencing delays in implementing its tobacco control policies and is presently increasing its production and, to lesser extent, processing of tobacco leaf. According to the WHO, agricultural land devoted to the harvesting of tobacco in Zambia increased over 350% in two decades from 1993 to 2013 [1]. The export of tobacco leaf increased from roughly $1.7 million in 1995 to $156.5 million in 2012 [2], although the figures vary depending on the source.^1^ While tobacco leaf as a percentage of total exports for Zambia is rather small,^2^ it is argued that tobacco is an important cash crop for farmers, about 10,000 of whom grow tobacco [3] and a significant source of employment in the country [4], although estimates of tobacco-related employment are contentious.

Zambia’s ratification of the WHO Framework Convention of Tobacco Control (FCTC) in 2008, however, creates policy incoherence within its government. Like most tobacco-producing countries that have ratified the FCTC, Zambia is now confronted with the need to scale back tobacco supply as articulated in Articles 17 and 18 of the Convention. Zambia is not alone in facing such policy incoherence [5–7], with policy mandates often localized and entrenched within the institutions that comprise differing government sectors, resulting in fragmentation [8] and/or silos [9, 10]. From this perspective, government is not monolithic but houses multiple institutional arrangements such as agencies, commissions, and working groups. It is in this context of departmentalism that Article 5 of the FCTC is put forward for governments to develop whole-of-government institutional mechanisms to coordinate implementation of the FCTC provisions [11]. In other words, the FCTC explicitly encourages intersectoral coordination of demand *and* supply reduction strategies across health, agriculture, trade, finance and all other implicated sectors. This type of whole-of-government approach results in new institutional challenges for tobacco control proponents. One of these challenges is the need for the health sector to develop working relationships with other sectors that pursue policies that support tobacco as an economic commodity [12, 13]. In doing so, tobacco control proponents must understand the institutional dynamics of these sectors, specifically the rules, norms and strategies [14–16] that perpetuate tobacco production, in order to shift these institutions towards supply reduction.

One way of promoting this understanding is to create an institutional context that is harmonized around health objectives while engaging sympathetically and meaningfully with the economic objectives of other sectors. To initiate such a process, Article 5.2 of the FCTC compels governments to “establish or reinforce and finance a national coordinating mechanism or focal points for tobacco control” [17]. A recent joint publication by the UNDP and the Framework Convention Secretariat found that, as of 2015, 90% of FCTC signatories in sub-Saharan Africa had a tobacco focal point within government [18]. Fifty-three percent of signatories in the region had created a national coordinating mechanism, a more complicated and involved institutional arrangement [18]. Such a mechanism would seek to bring together representatives from different sectors to establish dialogue around tobacco control. Not surprisingly, this can also bring to the surface deep tensions and divergences in mandates across the sectors [12, 19, 20], such as the case of the Philippines Interagency Committee – Tobacco where challenging conflicts emerged between the mandates of its ministries of trade and health in particular [12].

Deliberate and carefully conceived coordination among, and within, different sectors is a fundamental component of a whole-of-government approach to tobacco control, as envisioned by the FCTC. It is seen as vital to policy coherence [8, 21, 22], can be more cost-effective [23], and may ultimately promote financial, performance and political accountability [24, 25]. Christensen and Laegreid [9] explain that, while coordination is ideal, it does not come naturally. Government departments can be rigid and have a tendency towards minimal collaboration across sectors, resulting in inefficacies and internal conflicts. A tobacco policy implemented through a whole-of-government approach attempts to move ministries and agencies out of their silos. Given the historically entrenched and opposing tobacco mandates of economic and health sectors that persist in many countries, particularly those with weak health sectors, work to overcome such opposition will require a deep understanding of the institutional factors that shape and perpetuate their differing mandates. In other words, institutional analysis can contribute greatly to understanding the rules and organizational culture that shape the practice of government.

Drawing from qualitative interviews with key informants, this paper identifies, articulates and analyzes the institutional context governing tobacco production in Zambia. The objective of this analysis is to provide insights into the main barriers to implementing supply side measures to tobacco control in the country and to stimulate a discussion of policy approaches to shift towards a whole-of-government approach to tobacco control. Our analytical approach draws from Ostrom’s and colleagues’ Institutional Analysis and Development (IAD) Framework, using its three elements of rules, norms and action situations to locate and guide our qualitative data analysis [15]. We consider institutional rules to be roughly synonymous with the mandates that prescribe the goals and set the boundaries for a given government ministry (sector). We infer norms by reference to how actors within ministries (institutions) rationalize, understand or interpret the rules that govern their actions, adopting a discursive intuitionalist perspective [26, 27], drawing from Schmidt’s concept of “coordinative discourse” (the important role played by institutional actors with the power to shape such discourse when there are multiple competing interests) to examine how ideas operate within government to justify and rationalize policy approaches [26].

## Methods

We conducted semi-structured interviews with key stakeholders (see Table 1) from all the key ministries (Health, Foreign Affairs, Trade and Industry, Agriculture, Finance) (*n* = 26). Governance of matters of public import such as tobacco is not limited to government actors, but must include civil society organisations and commercial actors [28]. As such, we also interviewed representatives from diverse inter-governmental organisations, spokespersons from civil society organisations with an interest in tobacco control, and officials from tobacco interests. Individuals were included based on the criterion that they were involved in tobacco at the policy level. This meant that we targeted informants who had worked on developing and implementing tobacco control policy (i.e. tobacco was part of the individual’s portfolio), were involved in governing tobacco production, export and development of a tobacco supply chain, or who intervened as civil society advocates around tobacco-related policy issues (see Table 1). We used purposive sampling [29], supplemented by snowball sampling [30]. In most cases, two investigators were present at each interview, one from the Zambian research team and the other an international investigator. All interviews took place in the workplace of the informants, or in a location of their choosing, and lasted between 30 and 120 min. All but one interview was recorded and transcribed verbatim. The one interview not recorded was summarized in note form by the investigators, who compared notes post-interview to ensure accurate recall. Interviews took place between April and December, 2014. Ethics approval for this study was obtained from the ethics review boards of the University of Ottawa, McGill University, Morehouse School of Medicine (American Cancer Society) and the University of Zambia. Transcripts were entered into NVivo qualitative software and analyzed using thematic coding. A second stage analysis was undertaken specific to the institutional context of informants, coding for rules (mandates), norms (rationales), and action strategies (practices). Two of the international researchers worked closely with the two Zambian researchers to verify the analysis.

**Table 1:** Key Informants

Sector/Institution	Number Interviewed	Identifier (Quotes)
Agriculture	2	AGRI
Commerce, Trade & Industry	3	IND
Foreign Affairs	2	FOR
Trade	2	TRADE
Health	2	MOH
WHO	1	WHO
COMESA	1	COM
Tobacco Board of Zambia	1	TBZ
Tobacco Association of Zambia	1	TAZ
Zambia Agriculture Research Institute	3	ZARI
Multi-Facility Economic Zone	1	MFAZ
Zambia Development Agency	1	ZDA
Tobacco Companies	3	TOB
Health NGOs	3	NGO

## Results

There are three categories of results where the clash of mandates, norms and practices emerge most strongly: tobacco production and the Zambian economy, tobacco control and Zambians’ health, and the prospects for alternative crops to tobacco. Unless otherwise cited, statements of ‘fact’ by our interviewees reflect their perceptions; only in some instances did we utilize other sources to comment on these perceptions.

### Tobacco production and the Zambian economy

Most of our informants’ references to mandates and norms concerned the economics of tobacco production in Zambia, which presently revolves largely around tobacco growing and processing of the leaf. As Zambia’s tobacco leaf production is largely for export, most participants considered tobacco to be particularly important in terms of foreign exchange revenues. Growing and processing the leaf is labour intensive, reportedly outpacing other crops’ labour inputs by a “factor of three to one” (TOB). Representatives from the tobacco industry have been particularly enthusiastic to promote unsubstantiated statistics on the tobacco sector’s employment profile. For example, one informant from a Zambian tobacco company estimated that the industry employs 16% of the population of the country (TOB), with numbers ranging from 120,000 farmers and farm family members (ZDA) to a commonly cited figure of 450,000 employed across the total industry (MOH) [31]. These data generate the dominant narrative that tobacco production generates both profits and revenues, contributes to GDP, and is an important source of employment and rural livelihoods. In marked contrast, the government’s own statistics and related research we have conducted empirically challenge these claims, finding that the total number of individuals in tobacco farming households likely numbers around 54,000, there is no tobacco manufacturing within the country, and the number employed in leaf processing or trading is likely fewer than 5000 [32]. Nonetheless, as our findings below indicate, the dominance of this narrative both drives, and is driven by, the mandates of the different institutions and sectors engaged in Zambian tobacco production.

In advancing a vision of Zambia becoming a ‘middle-income country’ by 2030, for example, the Ministry of Commerce, Trade and Industry references three industries that are fast growing due to increasing “domestic demand (food, beverages and tobacco)” (¶ 4.1.11), while explicitly acknowledging the importance of tobacco’s contribution to manufacturing value-add (¶ 4.1.26) [33].^3^ Considering that there is no tobacco manufacturing in the country, this “importance” can be best interpreted as aspirational. The Ministry’s overall mandate is “to facilitate and promote…a sustainable and globally competitive commercial, trade and industrial base” [34], with tobacco falling within the “agro-processing” priority of the Ministry’s industrial development goal. This goal includes strategies to encourage and incentivize foreign or domestic investment in tobacco manufacturing, including cigarette production (IND) [20]. The most recent example of this (October 2016) was a government request to Japan Tobacco International to set up such a cigarette manufacturing facility in Zambia’s Eastern Province, where much of the tobacco crop is grown [35]. The Ministry’s pro-tobacco stance is further reflected in the normative emphasis its informants gave to tobacco’s role in employment – “we have a good number of peasant farmers who are tobacco producers, creating direct and indirect jobs” (IND) – and to “the country itself, in terms of exports” and foreign exchange earnings (IND).

Similar sentiments were expressed by our informant with the Tobacco Board of Zambia, a statutory body under the Ministry of Agriculture with the responsibility to “regulate and promote production, marketing and processing of the crop, as well as [its] export” (TBZ). Our informant gave particular emphasis to how “the nation is getting a lot of foreign exchange from tobacco,” adding that this is “because almost 100% of it is exported [with] revenue from tobacco estimated at 500 billion kwacha^4^ per annum [US$96.2 million]” (TBZ). The Tobacco Board itself supports development of cigarette manufacturing in the country (whether for domestic consumption or export is not clear), the value addition of which its CEO recently argued “could…trickle down to…the tobacco growers” with “the potential to contribute to [overall] economic growth” [31]. Our Board informant also underscored that “there are certain areas in Zambia where our people depend solely on tobacco” (TBZ), reinforcing the livelihood norm. This was a view somewhat challenged by our informant from the Tobacco Association of Zambia (representing tobacco farmers) who, speaking as a tobacco farmer, countered that “if your investment is purely tobacco, you won’t survive” (TAZ). He went on to describe how tobacco, even if the primary income generator, is usually just one of several income-earning crops grown by farmers.

Unsurprisingly, the argument of state reliance on tobacco revenue advanced by these two government institutions was also made by one of our tobacco company informants, who estimated total revenue from tobacco in 2013 at US$213 million. This is likely an overestimate, since data from the Food and Agriculture Organization place the value in 2012 at US$98 million [1], similar to the figure provided by the Tobacco Board of Zambia, above. Our informant nonetheless contended that “when you list the crops in comparison [tobacco] is the highest” in terms of generating revenue (TOB). Such comments are indicative of how the perceived contribution of tobacco to Zambia’s economic development is an established norm for those engaged in the industry, albeit one based less on “the income point of view” than on the industry being “one of the greatest employers compared to others” (TOB). It also explains the inflated numbers used rather freely to actively sustain the dominant tobacco narrative, with its subsequent normative impact.

The employment argument was given central prominence by our informant with the Zambia Development Agency, a semi-autonomous institution with ties to the Commerce Ministry and with a charge “to foster economic growth and development by promoting trade and investment through [a] private sector-led economic development strategy” [36]. Like the Tobacco Board informant, our Agency informant commented on the large number of farmers and their family members who relied upon tobacco production, and also noted the importance of tobacco “in reducing poverty levels in Zambia” (ZDA).^5^ His reference to poverty reduction is curious. Many Zambian government ministries or institutions articulate a shared goal of promoting “socio-economic development” (COM), “sustainable economic and social progress” [37] (COMESA), “socioeconomic development” [38] (Health) and “sustainable agriculture, economic and social development” [39] (National Farmers Union). Noticeably absent from the Agency’s mandate is any reference to this *social* dimension of development, which seems to be clearly focused on the investment, trade and enterprise side of the development ledger [36]. Our informant went on to describe tobacco as one of the country’s key industries which, as part of the agriculture sector, “enjoys certain incentives”, such as duty-free imports of inputs (e.g. machinery, chemicals) and tax reductions [20].

The Common Market for Eastern and Southern Africa (COMESA) is an intergovernmental body promoting regional economic integration through trade and investment liberalization amongst its member countries. Its principle remit is “to promote regional value chains,” such that “a product produced in this country leaves and [then] comes back…so promoting those industrial loops and growth within the COMESA member states…that is what we are doing” (COM). Tobacco figures broadly within this agenda, but less as a regional value-chain strategy than as a national industrial strategy since many of its member countries are keen “not just [to] be exporting raw leaf but…to actually keep that leaf within the borders and then [to] manufacture the finished product for export or for consumption” (COM).^6^ The goal of increasing job creation through industrialization and value-adding to traded goods is shared by the Foreign Affairs Ministry, which represents Zambia at the World Trade Organization; it is understandable, then, that our informant similarly saw tobacco production in a positive light, not just for the [tobacco growing] peasants but as well as the country itself in terms of exports” (FOR).^7^

Although it is more common to find competing mandates and norms across different institutions, this can also occur within the same institutional sector. This was most apparent in our study with the Ministry of Agriculture which, as a health NGO informant pointed out, is responsible for promoting alternative crops to tobacco (as part of the Ministry’s stated mandate), but is also encouraging “farmers to grow more tobacco because it brings revenue to the Zambian Government” (NGO). Alongside its subsidiary Tobacco Board, however, the Agriculture Ministry actively promotes and financially supports tobacco farmers “through setting up of revolving funds, allocation of land to mostly peasant farmers and establishment of institutions such as the…Tobacco Training School” [31]. Such promotions are considered indirect supports to tobacco farming. As one of our Agriculture informants explained:

[We] do not get involved with smallholder farmers in terms of cash crops they produce if these value chains have been long standing. The value chains are so well established... tobacco happens to be one of these. In terms of [direct] interventions… we feel the private sector is taking care of that (AGRI).

This explanation derives from the Ministry’s encouragement of “public-private partnerships” with certain “agriculture companies which come under ‘agro’ schemes whereby they focus on one crop” (AGRI). Tobacco is one such crop, where support for inputs (e.g. seeds, fertilizers, herbicides) is frequently provided by tobacco companies in contractual arrangements made with individual farmers (so-called ‘contract farmers’). Unlike ‘independent farmers’, contract farmers are spared the upfront costs of beginning each season and are guaranteed a buyer for their crop (although not a price) when the season ends, in a supply management system that offers some security to tobacco farmers but also places them under the monopsonistic financial control of tobacco leaf companies and, ultimately, tobacco transnational companies. More recently, however, the CEO of the Ministry’s subsidiary Tobacco Board has urged the government to extend its Farmer Input Support Programme to tobacco growers [31].^8^

The boundary between tobacco and non-tobacco farm support is somewhat blurred, with one of our informants describing geographically situated “camps” where Ministry staff “bring new technology, promote irrigation and mechanization”. They are required to assist “any farmer within that location” (tobacco-growing or otherwise) as part of the Ministry’s goal “to improve productivity, because productivity is a major problem in our country” (AGRI). This support can extend to tobacco research (although informants with the Zambia Agriculture Research Institute explained that this is no longer the case), but more commonly to intervening around “issues of marketing and pricing, because along the way we have had situations where we felt the producers were being, you know, exploited or blacklisted by the buyers” (AGRI). Even as one of the Agriculture Ministry’s goals is to promote crop diversification, a senior Ministry official as recently as 2016 spoke out against any attempts to ban tobacco production while claiming that tobacco contributed “3% to the Gross Domestic Product” [40](REF), a figure an order of magnitude higher than World Bank and FAO estimates, 0.4% of GDP [1]. The official also repeated the normative refrain that any loss in tobacco production or trade would “worsen poverty in rural communities which depend entirely on tobacco production” and called for “dialogue between the tobacco sector and health authorities on the country’s adoption of the tobacco control treaty” [40].^9^

### Tobacco control and Zambian health

Although our informants spoke most extensively on tobacco’s economic importance, the issue of tobacco control and health received considerable attention. The Ministry of Health was identified by several informants as the “lead Ministry” for tobacco control. “Strengthening enforcement of legislation on tobacco use” is identified as a key intervention for the Ministry [38] (p.20), albeit one that has yet to be implemented; while tobacco use is seen rather singularly as a “lifestyle” factor [38] (p.18). There is no reference to the FCTC, or to tobacco farming or production, in the Ministry’s 2011–2015 strategic plan, although the ministry is scheduled to introduce a new plan sometime in 2017. One of our health informants did state that the Ministry’s policy directorate was requested to develop “legislation following [Zambia’s ratification of the] WHO FCTC”. Our informant with the WHO country office also noted that it is the Health Ministry’s responsibility to implement “procedures and [advance] the process of accelerating the enactment of the [FCTC] law” (WHO). The WHO Country Office in Zambia, in turn, is mandated to perform an advisory function, “providing financial and technical support to government, to assist government to domesticate the FCTC, so they can implement the supply and demand reduction measures which are in the [Convention] by specifically ensuring that they come up with a domestic law which captures the major supply and demand measures which they are obliged to implement” (WHO). This advisory role includes low-key advocacy efforts, such as writing to ministers or convening regional meetings to prod “those countries that have yet to implement these laws” (WHO).

Our Health Ministry informants also spoke of friction among the different Ministries and their competing mandates. The Ministry had attempted to involve the Ministry of Finance in a commitment to increase taxes related to tobacco products (one of the reduction strategies advanced by the FCTC), but Finance did not engage. Further attempts were made to develop inter-ministerial collaboration among the Commerce and Agriculture Ministries, Zambia’s Bureau of Standards, and the Zambia Revenue Authority (MOH), but continually faced challenges because the mandates and priorities of these other institutions conflicted with those of Health. Our informants further described how the Ministry had received comments on the Australia Plain Packaging Proposal from Foreign Affairs, which argued that provisions in the proposal may contradict other laws in Zambia (e.g. the Zambia Competition and Consumer Protection Act). This Act states that the laws put in place in Zambia should not interfere with the running of businesses. If the plain packaging law were to pass, it would mean consumers would be disadvantaged as they wanted to know the brand available. Foreign Affairs further advanced that the World Trade Organization (WTO) would not allow Health to enact laws that interfered with trade (MOH), a statement which is not actually true except under certain conditions.

These mandate and normative conflicts with tobacco control goals, however, were most pronounced with Commerce and Agriculture:

Our line Ministry, we have had challenges with Commerce, and their concern is that we are getting money out of tobacco and it’s assisting the government…so [while] we are worried about the health of the people, our friends the agronomists [in Agriculture] are worried about the crop and the industry [Commerce] is worried about business. It’s a triangle (laughs) (MOH).

The norm from within the Ministry was that health staff were unavoidably trapped within this triangle, since “the problem is because we know that just as much as we want our people to be healthy, we also need money to procure drugs, and where is this money coming from?” (MOH) the answer to the rhetorical question being from government revenues relying, in part, on tobacco. Health NGOs in distinct contrast were more dismissive of this concern, stating bluntly that “the government is spending more on treatment [for tobacco diseases] than the revenue they are getting from…tobacco” (NGO).

To treat a cancer patient it takes a lot of money, it’s expensive. So whatever revenue the Zambian government is getting…from the tobacco sales and the tobacco industry, it goes to Ministry of Health to treat the cancer patients (NGO).

Another informant from the Health Ministry seemed to accept this broad brush assessment, noting that “we have these [employment and revenue] challenges but we should also look at the negative impact [tobacco] has on the human health and…always say that even if that money is made, it still ends up in procuring medicines for people [suffering tobacco diseases] or rehabilitating them or whatever” (MOH). These statements invert the dominant pro-tobacco norm (that it creates a positive economic externality) by contending that it actually risks being an economic drain, an argument given broad support by the World Bank report “Curbing the Epidemic” in 1999 [41]. While such an assertion is reasonable given the mandate of the Health Ministry (and other health NGOs) to improve and protect health, it rests on demonstrating that smoking rates and tobacco-related diseases are sufficiently prevalent in Zambia that they offset tobacco’s economic benefits.^10^

These pro-tobacco economic arguments were ubiquitous, reflected long-standing arguments advanced by tobacco transnational companies, and led many informants to rationalize the continued or expanded production of a good that many openly acknowledged was known to cause health problems. Several informants reconciled this normative dissonance by claiming that “It’s not the Zambian who consumes the tobacco” (AGRI), citing the fact that most of Zambia’s tobacco is exported, primarily to China. A tobacco company informant similarly stated that, because “Zambians don’t smoke” the government shouldn’t try to convince to stop growing tobacco by arguing that “we lose money to health costs due to consumption” (TOB). One informant further linked his assumption of low smoking rates of Zambians with the country’s ambivalence towards the FCTC and new tobacco control measures:

I think in Zambia we don’t have a very big tobacco problem in terms of the number of people that smoke... if Zambians are not heavily smoking they are not really interested in the purpose and implementation of the FCTC (FOR).

While most informants indicated domestic consumption of cigarettes to be low, statistics show otherwise, with 24% of Zambian males reported to smoke in 2013 (WHO, 2016).

An informant involved in the trade side of the Foreign Affairs emphasized the importance of striking “a balance between the fiscal issues [the foreign exchange tobacco trade generates] as well as the health issues” (FOR). Even when recognizing that “tobacco is harmful,” there is a need to “balance that [with] the need to get to a certain level of economic development” (COMESA); that “much as the [FCTC] proposal…with health issues might make sense, we also need to ensure that we do not disadvantage the farmers who are involved in production of tobacco” (TRADE). Other informants were more explicit:

[The FCTC] was a threat to tobacco producing countries…and that regulation might affect tobacco-producing countries in terms of revenue…All member states were in agreement…that we should…not entertain this kind of regulation, which threatens the economies of SADC [Southern African Development Community] and tobacco production (FOR).

The strategy of creating country coalitions in support of rural tobacco livelihoods and against tobacco control measures, which derives more from norms than from institutional mandates, has been reported on in other findings from our research study [20, 42]. Our COMESA informant similarly noted how, a year before we began our study, British American Tobacco lobbied COMESA member countries to issue a joint position opposing the FCTC because it risked “reducing consumption of tobacco” negatively impacting the demand for tobacco leaf (COM). Tobacco company arguments were always on tobacco’s “contribution to GDP…that is the foundation” (COM). In a more normative vein, our informant thought that Zambia needed first to grow its economy before tackling tobacco’s health issues, “because it seems the more prosperous countries are [the ones] dealing with tobacco” (COM). This appeared to be shared by one of our Foreign Affairs informants who argued that, for the moment at least, “engaging in treatment [to reduce] tobacco use” may have to be considered “a luxury” (FOR). For both informants, GDP growth appears to be more important than either supply side measures, or even domestic tobacco use reduction.

Others were less concerned with rationalizing the health/tobacco nexus, and simply retreated to their institutional mandate. When two of our informants from Commerce were asked about the direct contradiction in their tobacco promotion goals with those of the Ministry of Health, they simply restated that their role was “to promote industrial development” (including tobacco manufacturing), and that any health concerns related to tobacco were the responsibility of the Health Ministry. They also invoked the ‘personal choice’ norm, that smoking was a strictly private affair outside of the realm of the state (IND), an argument made by several other informants who talked about education rather than regulation to curb Zambian smoking rates. In response to a question about the FCTC’s suite of advertising and sponsorship bans, warning labels and taxation measures, our Tobacco Association informant countered, “Let me again mention that [these] are punitive measures [whereas] I believe in education, through talking to people, not glorifying [smoking]” (TAZ). He also raised the ‘smoker’s rights’ argument advanced by tobacco transnationals, stating that “if you are driving and you want to light a cigarette, then light it. It is my choice to get out if I want to [to avoid you smoke]” (TAZ).

It was the ‘balance’ norm, however, that predominated amongst informants in sectors engaged in the tobacco industry or its promotion. The strength of this same norm was further evident in how it was also invoked by those with only peripheral engagement with tobacco production, or charged with implementing tobacco control measures. One of our health informants, for example, accepted completely the tobacco employment and revenue argument:

We have the employment offered by the industry. We have the revenue from the industry and [we] cannot just…throw it away, because the government gets revenue from the same tobacco (MOH).

While health informants were well versed in the economic arguments for tobacco, tobacco informants were less well versed in the health concerns. Despite a health informant stating that, because the FCTC was signed by Zambia’s President, “all of us as members of the government are supposed to listen to it” (MOH), several informants from other Ministries claimed they had never heard of the Convention, or were unaware of its requirements, which include proscriptions on governments’ support for or encouragement of tobacco production.^11^ One of our Foreign Affairs informants was under the mistaken impression that Zambia was still negotiating ratification of the FCTC when in fact the country had ratified the Convention 5 years earlier, and that it was Foreign Affairs that led the FCTC negotiations. He also didn’t perceive any conflicts between the FCTC and the mandates of other Ministries, arguing that if they were the lead (or ‘line’) Ministry (in the case of the FCTC, Health) would convene meetings with other affected Ministries (including his own) to iron them out:

But when an issue has little controversy in terms of the running … it’s easy to let the line ministry run with it. In my understanding we are not having [controversial issues] at the moment. So I assume the implementation of the Convention is going smoothly (FOR).

Others were dismissive of the Convention, regarding it as the sole prerogative of the Health Ministry, as one informant from Agriculture expressed:

They [health] must have signed those [FCTC] protocols on behalf of maybe the country (laughs)…In the meantime we are the people who are actually involved in the production and… you guys, you sign for this, you don’t even consult us (AGRI).

One of our tobacco informants was blunter, stating that the leaf transnational for which he worked:

…is not opposed to the FCTC…but to the way tobacco is looked at. There is nothing good in it [the FCTC] about [tobacco] but there is good things about tobacco when you look at it on the economic side. [We] think the FCTC is too extreme (TOB).

### Alternatives to tobacco production

One of the supply-side tobacco control strategies advanced in the FCTC, and another theme probed in our study, is to promote alternative livelihoods for tobacco farmers. An Agriculture informant noted that crop diversification “is part of our mandate, yes, we are now talking about diversification…we have a fully-fledged department, which is there just to assist smallholder farmers” (AGRI). The diversification effort, however, is not specific to tobacco but is part of a broader Ministry mandate to “increase the value and growth rate of crop exports” with an emphasis on “domestic and international markets” regardless of crop [43] (p.6). Tobacco is a concern only insofar as there is worry that global and domestic consumption might start to decline “due to health issues” [43] (p.17). The Zambia Development Agency also takes a generic approach to diversification, “not saying that farmers who are growing tobacco [should] grow other products, what we are doing is looking at other products that we encourage [all] farmers to grow” (ZDA).

Working on tobacco alternatives forms part of the Health Ministry’s work, but there is the pervasive “challenge in that we have to convince the industry and the government that whatever we do [we] will have to find a system that is going to replace what we have now as a source of revenue” (MOH).

The farmers are saying, if you find a crop that is as marketable or ready as an alternative crop that will…give them income they will definitely shift. So the challenge we have is to find the crop that is as marketable, or even more marketable, than tobacco (MOH).

This challenge is inherently (or should be) “whole-of-government,” as the WHO informant acknowledged: “These are important economic policies where you need multi-sectorial approaches because the Ministry of Health alone cannot manage to support these farmers, help them to go into alternative crops” (WHO), a key Ministry partner in this being Agriculture. However, to the contrary, there is a more prominent institutional initiative in place to foster processing and manufacturing of agricultural crops through investment incentives. This initiative treats tobacco as any other crop and our findings have pointed out that this has generated interest in tobacco processing and manufacturing, with the potential to lead to greater demand for tobacco growing within the country [20].

Our Agriculture informant acknowledged that the government “should now be seen to discourage tobacco growing and maybe assist farmers growing tobacco to diversify to crops which are not as harmful” but added that “the alternatives are not forthcoming” (AGRI). As other Agricultural informants expressed, “the [tobacco] sector feels they need to be given a bit more time to come up with an exit strategy” (AGRI). These perceptions reflect another tobacco norm that, as our informant with the Tobacco Alliance, expressed: “Tobacco will pay for the development, and then that eventually gives the farmer the ability to decide to diversify into other crops” (TAZ). While all informants emphasized the importance of working with tobacco farmers themselves around the identification of crop alternatives, those representing the industry were more emphatic that “where there are issues of finding alternative crops for tobacco…this is supposed to be voluntary, the farmers themselves they will see that this crop is better than tobacco. It’s not something that you can force the farmers to do” (TBZ).

The government-funded Zambia Agriculture Research Institute, perhaps reflecting its mandate to identify alternative crops to tobacco, gives a somewhat optimistic spin to that possibility: “Crops like soya beans, if properly managed and grown, could compete favourably with tobacco” (ZARI). Although hedging this claim somewhat, “I can’t say with confidence that’s the case, I think those are some of the studies we need to carry out to be certain” the ZARI informant nonetheless noted that “soya beans is a very good alternative to tobacco…and for us, we look at the entire value chain” (ZARI). Reference was made to a new soya processing plant^12^ that would cater to small-scale farmers (previously a barrier for entering the soya market) and that, like tobacco processing facilities, would pay immediate cash: “You go in with soya beans, you walk out with cash” (ZARI). The absence of GMO soya would also ensure a market niche for Zambian soya exports, with the ZARI informants commenting that, with tobacco production on a decline, “the increase in soya production has been dramatic.” The challenge is “to change the crop culture, especially in those areas where farmers have been growing tobacco…to demonstrate [that soya] is a good alternative crop” (ZARI).

## Discussion

Returning to our theoretical frame of mandates, norms and practices, a repetitive theme coming through the interviews was that each institution or Ministry was responsible for only one small segment of Zambia’s tobacco industry. The majority of those interviewed frequently referred to another unit or Ministry when a question appeared to take them away from their core area of work, explaining that the extent of their mandate ended ‘here’ and that it would be the responsibility of another to take it from ‘there’. When asked if it promoted the export of alternative crops to tobacco, the Foreign Affairs ministry responded, “That will be the Ministry of Agriculture” (FOR). On the FCTC, the SADC Unit in Foreign Affairs “…threw it to Commerce and ever since it has not come back to SADC meetings” (FOR). However, those institutions who were responsible for tobacco supply (production) clustered around common dominant narratives. These narratives tied tobacco production to wider economic development discourses, particularly in terms of the necessity of tobacco for the country’s overall development. The discursive nature of these practices is important to highlight, in that we can see patterns of argumentation tied to widely accepted and seemly implicit and taken for granted ideas about the nature of economic development. Although this finding has interesting theoretical implications pertaining to the role of ideas in what Schmidt has termed coordinative discourse [23, 24], these findings also have practical implications for how we think about and act for institutional change. These findings point out that there is a reciprocal dynamic between ideas and structures, and that attention to structural change alone may not be sufficient to improve policy coherence.

There have been wide calls for the establishment of intersectoral mechanisms to ensure that the FCTC is implemented across government, the logic being that such a mechanism will facilitate rule-formation and that the structural intervention will enlist support from non-health sectors for tobacco control goals. However, this study demonstrates that non-health sectors are not simply neutral bureaucratic institutions but are deeply invested in particular ideas of government and development. From this perspective, ideas become a target for intervention in addition to structural interventions. In other words, “rules-based” interventions are not a total solution but rather a part of a broader strategy to shift norms and corresponding strategies. The case of Brazil is illustrative of both the potential of such high-level commitment to generate attention to tobacco control across sectors and the difficulty of reconfiguring norms that foster tobacco production. In 1999, the President of Brazil mandated the creation of CONICQ, a “whole-of-government” body to facilitate the negotiation and implementation of the FCTC. The fact that the body was established by Presidential decree brought legitimacy to an intersectoral approach to tobacco control and established the basic structure for intersectoral cooperation [44]. Despite this high-level policy directive, general departmental mandates oriented towards economic development were perpetuated by pro-tobacco norms and fostered strategies that supported tobacco production within the country [45, 46].

Practice rigidities deriving from competing Ministerial mandates are not uncommon, but what is striking in our findings was less that there were such conflicts, but more the near universal diffusion of pro-tobacco norms arising from the mandates of a few key Zambian Ministries (e.g. Commerce, Agriculture, Foreign Affairs). Although we are focused in the domain of tobacco, the “pro-tobacco” discourses appear to be largely tied to overarching development discourses. In other words, a particular version of “economic development” is universally espoused. This version of economic development, while occasionally referencing GDP growth, leaves its rationale largely implicit; informants did not engage with this economic development discourse in a reflexive manner but rather championed a particular narrative of the economic necessity of tobacco for the economy of Zambia. From this view, it is perhaps not surprising that these norms infiltrated the perceptions of those in the Health Ministry and health NGOs to a much greater extent than any health norm appeared to temper the tobacco/economic rationales. This case demonstrates that economic norms dominate the government landscape thus ordering the operations of government across sector around these norms. Although these norms are not static, our findings suggest that, like other theoretical and empirical work on the hegemony of a neoliberal economic order (e.g. [47–49]), they are indeed deeply entrenched in the ideas and practices of government.

From a practical perspective, these findings point to patterns of arguments that can be targeted to refute their factual basis. Although facts are only part of wider discursive patterns that shape institutions, they have the potential to resonate with decision-makers and start to shift discourses. The first of these may well be inverting the economic argument by creating much more awareness of the extent of cigarette use already in Zambia, that certain government Ministries accept its growth almost as a given (desirable or otherwise), and that the costs of treating tobacco-related diseases may well offset the public revenues earned from the tobacco industry.

A second resides in the debatable role of tobacco in contributing to poverty reduction. The data on poverty in Zambia do not support this, apart from demonstrating a counterfactual that if tobacco production did not exist, the rise in poverty rates would have been even greater; and our own study of tobacco farmers’ incomes, which accounts for the value of family labour in what is one of the most labour intensive agricultural crops [32], and questions how essential this crop may be for rural livelihoods.

This second normative reversal is buttressed by a third: the findings from ZARI that soya may well be a more (or at least equally) lucrative crop for small-hold farmers presently habituated to tobacco production.

A fourth way into challenging the dominant tobacco/economic discourse is a growing acceptance that tobacco is a ‘dying’ industry, as concerns over health effects slowly gain broader traction, with even tobacco proponents considering a future time when people no longer smoke.

A return to Ministerial mandates may, in fact, help to speed things along, with the frequent elision of the ‘social’ with the ‘economic’ in many Zambian government sectors providing a basis for developing a more intersectoral approach to managing the tension between tobacco co-existing simultaneously as both an economic commodity and as a health hazard. This was noted by several of our informants, although whether this leads to the type of intersectoralism needed to promote a more tobacco-free Zambia remains to be seen. There is some cautious optimism, however, with the Zambian government in its final report to the UN General Assembly on the Millennium Development Goals acknowledging that “the “working in silos” approach to tackling developmental challenges by sectors has had a negative impact on addressing the mutually reinforcing factors required to address the [MDGs].”

In order to redress this, Government has, in the Revised Sixth National Development Plan, put in place an intersectoral collaborative framework to promote synergies between various sectors in the implementation of national development plans [and] to promote collectiveness in tackling development interventions between sectors [50].

## Conclusion

Tobacco leaf production and export are growing in Zambia, while the normative claim of its importance as a source of employment and revenue persists. In 2008, Zambia ratified the FCTC which requires Zambia to scale back production of tobacco. The Ministry of Health bears the principle responsibility to oversee the implementation of the FCTC’s provisions. However, there are many institutional actors in Zambia’s tobacco sector, each with their own mandates and priorities, some of which conflict with those of the Ministry of Health and the provisions of the FCTC. Acknowledging the reality of institutional departmentalism, the FCTC calls for governments to develop a whole-of-government approach to coordinate implementation of FCTC provisions. Zambia has not yet succeeded in coordinating such an approach or establishing functioning intersectoral mechanisms to advance FCTC compliance. There are, however, several recent shifts in normative discourse, policy and research findings that, if well leveraged, could increase intersectoral implementation of the FCTC. These shifts include the increasing number of countries adopting plain packaging laws, the loss by tobacco transnational companies using trade and investment treaty provisions to challenge tobacco control measures in cases against Uruguay and Australia, the inclusion of partial carve-outs of tobacco control measures from investment rules in new free trade agreements (such as the revised Trans-Pacific Partnership agreement, awaiting signature), and economic analyses of tobacco farming livelihoods conducted as part of our larger set of research studies involving our three African countries, that refute the economic livelihood argument that underpins much of the pro-tobacco economic arguments [51].

## Abbreviations

COMESA: Common market for eastern and Southern Africa
FCTC: Framework convention of tobacco control
IAD: Institutional analysis and development (Framework)
MDG: Millennium development goal
SADC: Southern African development community

## Footnotes

1 Trade value in US$.

2 Tobacco leaf accounted for only 1.67% of the total of Zambia’s exports in 2012.

3 In a revealing aside, the same report comments with respect to trade that when Zambia liberalized in 1993 as a conditionality of a structural adjustment loan, “The immediate impact…was a further deterioration in manufacturing sector performance” (¶ 4.1.10). One rebounding exception in the later 1990s was growth in the tobacco sector.

4 This estimate uses the old ZMK currency, and not the post 2013 re-valued ZMW.

5 This is a questionable assertion. Over the period that tobacco production increased in Zambia (since the late 1990s), the World Bank extreme poverty rate (<$1.90/day in consumption) increased from 41% (1996) to 64% of the population (2010), the latest year for which data are available (data adjusted to 2011 prices). There was also a parallel decline in the share of income held by the lowest 20% since the early 2000s. World Bank Indicators, http://data.worldbank.org/country/zambia, Accessed January 10, 2017.

6 We explore how regional trade agreements shape tobacco production and consumption patterns in a separate paper presently under review.

7 The individual also seemed to be under the mistaken impression that Zambia was still negotiating ratification of the FCTC when in fact it had ratified the convention 5 years earlier.

8 This support program for small-scale farmers in 2015 was expanded to include nine crops (maize, rice, sorghum, groundnuts, orange maize, soya, cotton, beans and sunflower) but continues to exclude tobacco (http://www.parliament.gov.zm/sites/default/files/images/publication_docs/Ministerial%20Statement%20-%20Hon.%20Minister%20of%20Agric%20and%20Livestock%2001.10.15.pdf) Accessed 10 January 2017.

9 Such dialogue is actually discouraged by the FCTC’s Article 5.3, which requires that “in setting and implementing their public health policies with respect to tobacco control, Parties shall act to protect these policies from commercial and other vested interests of the tobacco industry in accordance with national law.”

10 Neither the Ministry of health NGO informant offered any substantiation for their perceptions. In 2015 Zambia committed 9.6% of its expenditures to health, four times what it provided in the Farmer Input Support Program. In US$ terms, total health spending (US$628 million) was around 7 times more than annual revenues earned from tobacco, although what portion of health spending went to tobacco-related diseases is not known. See: http://www.parliament.gov.zm/sites/default/files/images/publication_docs/2015%20Budget%20Speech_0.pdf Accessed 10 January 2017.

11 This requirement is stated as one of the principles of Article 5.3 of the Convention (http://www.who.int/fctc/guidelines/article_5_3.pdf Accessed 9 January 2017).

12 Recent media accounts suggest there may be more than one, with a new one opening as recently as December 2016 in Zambia’s Northern Province (https://www.lusakatimes.com/2016/12/07/kasama-another-agro-processing-plant/ Accessed 8 January 2017).

## References

[1] FAO. FAO Statistics Database. Food and Agriculture Organisation. 2015. http://www.fao.org/faostat/en/#datahttp://www.fao.org/faostat/en/#data.

[2] WHO (2016). Tobacco agriculture and trade.

[3] Tembo S, Sitko N (2013). Technical compendium: descriptive agricultural statistics and analysis for Zambia.

[4] Namutowe J. Zambia: Tobacco Industry - Zambia’s Emerging Income Earner. Times of Zambia. 2013. http://allafrica.com/stories/201307310638.htmlhttp://allafrica.com/stories/201307310638.html.

[5] Collin J (2012). Tobacco control, global health policy and development: towards policy coherence in global governance. Tob Control.

[6] Butler S (2009). Obstacles to the implementation of an integrated National Alcohol Policy in Ireland: nannies, neo-liberals and joined-up government. J Soc Policy.

[7] Axelsson R, Bihari-Axelsson S (2005). Intersectoral problems in the Russian organisation of public health. Health Policy..

[8] Kavanagh D, Richards D (2001). Departmentalism and joined-up government. Parliam Aff.

[9] Christensen T, Lægreid P (2007). The whole-of-government approach to public sector reform. Public Adm Rev.

[10] Christensen T, Lægreid P (2008). The challenge of coordination in central government organizations: the Norwegian case. Public Organ Rev.

[11] World Health Organization. Guidelines for implementation of Article 5.3 of the WHO Framework Convention on Tobacco Control. Geneva: World Health Organization. http://www.who.int/fctc/guidelines/article_5_3.pdf.

[12] Lencucha R, Drope J, Chavez JJ (2015). Whole-of-government approaches to NCDs: the case of the Philippines interagency committee—tobacco. Health Policy Plan.

[13] Drope J, Lencucha R (2013). Tobacco control and trade policy: proactive strategies for integrating policy norms. J Public Health Policy.

[14] Ostrom E (1986). An agenda for the study of institutions. Public Choice.

[15] Ostrom E (2011). Background on the institutional analysis and development framework. Policy Stud J.

[16] Ostrom E (2005). Understanding institutional diversity.

[17] WHO. WHO Framework Convention on Tobacco Control. World Health Organization. 2003. http://apps.who.int/iris/bitstream/10665/42811/1/9241591013.pdf?ua=1&ua=1.

[18] Drope J, Lencucha R, Magati P, Small R (2016). Tobacco control governance in sub-Saharan Africa: implementing article 5.2(a) of the World Health Organization framework convention on tobacco control.

[19] Brusca I, Montesinos V (2009). International experiences in whole of government financial reporting: lesson-drawing for Spain. Public Money Manag.

[20] Lencucha R, Drope J, Labonte R, Zulu R, Goma F (2016). Investment incentives and the implementation of the framework convention on tobacco control: evidence from Zambia. Tob Control.

[21] Greer SL, Lillvis DF (2014). Beyond leadership: political strategies for coordination in health policies. Health Policy.

[22] Duit A, Galaz V (2008). Governance and complexity—emerging issues for governance theory. Governance.

[23] Vangen S, Huxham C (2003). Enacting leadership for collaborative advantage: dilemmas of ideology and pragmatism in the activities of partnership managers. Br J Manag.

[24] Wilkins P (2002). Accountability and joined-up government. Aust J Public Adm.

[25] Brinkerhoff DW (1996). Coordination issues in policy implementation networks: an illustration from Madagascar’s environmental action plan. World Dev.

[26] Schmidt VA (2008). Discursive institutionalism: the explanatory power of ideas and discourse. Annu Rev Polit Sci.

[27] Schmidt VA (2010). Taking ideas and discourse seriously: explaining change through discursive institutionalism as the fourth “new institutionalism.”. Eur Polit Sci Rev.

[28] Stoker G (1998). Governance as theory: five propositions. Int Soc Sci J.

[29] Miles MB, Huberman AM. Qualitative Data Analysis: An Expanded Sourcebook. London: SAGE; 1994. p 358.

[30] Noy C (2008). Sampling knowledge: the hermeneutics of snowball sampling in qualitative research. Int J Soc Res Methodol.

[31] Zambia: Tobacco Vital to Economy. Times of Zambia. 2015. http://www.times.co.zm/?p=57328.

[32] Goma F, Drope J, Zulu R, Li Q, Chelwa G, Labonte R (2016). The economics of tobacco farming in Zambia.

[33] Vision 2030: A prosperous middle-income country nation by 2030. Lusaka, Zambia: Republic of Zambia; 2006.

[34] Ministry of Commerce, Trade and Industry. Zambia: Republic of Zambia; 2017. Available from: http://www.mcti.gov.zm/index.php/about-mcti/about-mcti. Accessed 6 May 2017.

[35] Government requests JTI to set up a cigarette manufacturing plant in East. Lusaka Times. 2016 Oct 13; Available from: https://www.lusakatimes.com/2016/10/13/govt-requests-jti-set-cigarette-manufacturing-plant-east/. Accessed 6 May 2017.

[36] ZDA. Background [Internet]. Zambia: Zambia Development Agency; 2017. Available from: http://www.zda.org.zm/?q=content/background. Accessed 6 May 2017.

[37] COMESA. COMESA Vision and Mission [Internet]. Lusaka, Zambia: COMESA; 2017. Available from: http://www.comesa.int/comesa-vision-and-mission/. Accessed 6 May 2017.

[38] Ministry of Health. National Health Strategic Plan 2011–2015. Lusaka, Zambia: Ministry of Health; 2010.

[39] About ZNFU. Zambia: Zambia National Farmers Union; 2017. Available from: http://www.znfu.org.zm/about_us. Accessed 6 May 2017.

[40] Zambia refuses to ban tobacco production. News Ghana [Internet]. 2016; Available from: https://www.newsghana.com.gh/zambia-refuses-to-ban-tobacco-production/. Accessed 6 May 2017.

[41] Jha P, Chaloupka FJ. Curbing the Epidemic: Governments and the Economics of Tobacco Control. Washington: World Bank Publications; 1999. p 140.

[42] Lencucha R, Drope J, Labonte R (2016). Rhetoric and the law, or the law of rhetoric: how countries oppose novel tobacco control measures at the World Trade Organization. Soc Sci Med.

[43] The National Agriculture Policy 2012-2030. Zambia: Minstiry of agriculture and co-operatives. p. 2011.

[44] Lee K, Chagas LC, Novotny TE (2010). Brazil and the framework convention on tobacco control: Global Health diplomacy as soft power. PLoS Med.

[45] Lencucha R, Drope J, Bialous S, Richter AP, Silva V da Costa e. Institutions and the implementation of tobacco control in Brazil. Cadernos de Saúde Pública. 2017;33(Suppl. 3):e00168315. https://dx.doi.org/10.1590/0102-311x00168315. 10.1590/0102-311X00168315 29069213

[46] Bialous S, da Costa E Silva V, Drope J, Lencucha R, McGrady B, Richter AP. The Political Economy of Tobacco Control in Brazil: Protecting Public Health in a Complex Policy Environment. Rio de Janeiro, Brazil: Centro de Estudos sobre Tabaco e Saúde, Escola Nacional de Saúde Pública/FIOCRU; 2014.

[47] Harvey D (2003). The new imperialism.

[48] Labonté R, Stuckler D (2016). The rise of neoliberalism: how bad economics imperils health and what to do about it. J Epidemiol Community Health.

[49] Schrecker T, Bambra C. How Politics Makes Us Sick: Neoliberal Epidemics. London: Springer;2015. p 179.

[50] Kalamwina C (2015). Managing the transition from the millenium development goals to the sustainable development goals: what will it take (report to the United Nations).

[51] Makoka D, Drope J, Appau A, Labonté R, Li Q, Goma F (2017). Costs, revenues and profits: an economic analysis of smallholder tobacco farmer livelihoods in Malawi. Tob Control.

